# Ewing sarcoma of the pancreas: a pediatric case report and narrative literature review

**DOI:** 10.3389/fonc.2024.1368564

**Published:** 2024-04-17

**Authors:** Zhensheng Liu, Jian Bian, Yong Yang, Decheng Wei, Shiqin Qi

**Affiliations:** Department of General Surgery, Anhui Provincial Children’s Hospital, Hefei, Anhui, China

**Keywords:** pancreatic neoplasms, child, Ewing sarcoma, small round cell malignant tumor, pancreaticoduodenectomy

## Abstract

Ewing’s Sarcoma (ES) is an rare, small round-cell sarcoma that predominantly occurs in children and young adults, with both skeletal and extraskeletal manifestations. However, pancreatic ES, due to its rarity, is infrequently featured in scholarly literature, with only a scant 43 reported instances. Our study describes a case of pancreatic ES in an 8-year-old boy who was found to have an abdominal mass. Following an exhaustive examination, the boy was diagnosed with a neoplasm in the pancreatic head and underwent a complex surgical procedure encompassing pancreatoduodenectomy and partial transverse colectomy. Immunohistochemical assays confirmed the neoplastic cells’ positivity for Cluster of Differentiation 99(CD99), Vimentin, and NK2 Homeobox 2(NKX2.2), while genomic testing identified an EWSR1-FLI1(Ewing Sarcoma Breakpoint Region 1–Friend Leukemia Integration 1) gene fusion. This led to a conclusive diagnosis of pancreatic Ewing’s Sarcoma. The patient underwent seven cycles of adjuvant chemotherapy, alternating between VDC (Vincristine, Doxorubicin, Cyclophosphamide) and IE (Ifosfamide, Etoposide) tri-weekly, but did not undergo radiotherapy. At present, the patient remains neoplasm-free. Through our case analysis and comprehensive review of the existing literature, we aim to underscore th rarity of pancreatic Ewing’s sarcoma and to highlight the efficacy of our individualized therapeutic approach.

## Introduction

Ewing sarcoma is characterized by a unique, small, round-cell sarcoma that exhibits gene fusions, typically involving one element from the FET family of genes, commonly Ewing Sarcoma Breakpoint Region 1 (EWSR1), and an element from the E-twenty-six (ETS) protein family of transcription factors. The most common Ewing sarcoma translocation, which is present in approximately 85% of cases, is t (1, 2)(q24;q12), leading to the EWSR1-FLI1 (Ewing Sarcoma Breakpoint Region 1–Friend Leukemia Integration 1) fusion transcript (3). The use of terms such as “Askin tumor” and “primitive neuroectodermal tumor (PNET) “ is not recommended according to the 2020 World Health Organization’s (WHO) classification of undifferentiated small round cell sarcomas of bone and soft tissue to prevent potential confusion or misunderstanding (3). ES generally originates in bones and soft tissues, but there have been occasional reports of these tumors arising in unconventional locations such as the gastrointestinal tract, genitourinary tract, adrenal glands, parotid glands, and lungs (4–6). Apart from the aforementioned organs, such tumors could potentially emerge in the pancreatic region, corresponding to nearly 0.3% of primary pancreatic neoplasia occurrences (6, 7). Grasping the diverse presentation of ES and the various anatomical sites where it could potentially develop is crucial for clinicians to make timely diagnoses and develop appropriate management strategies for cases involving atypical locations (8).

This investigation presents a comprehensive analysis of a rare instance of pancreatic Ewing’s sarcoma in an eight-year-old boy, exploring the diagnostic methodologies and therapeutic approaches utilized along his clinical journey. Through this research, we aim to enhance our understanding of rare tumors, improve early diagnosis, and develop more effective management strategies for such complex cases.

## Case description

An 8-year-old boy was admitted to the pediatric general surgery department due to a palpable mass in the upper abdominal region, with no prior family history of cancer. Upon physical examination, no obvious enlargement of the superficial lymph nodes was palpated throughout the body. However, in the right upper quadrant of the abdomen, a localized protrusion revealed a firm, non-tender mass upon palpation. This mass, approximately 12cm x 10cm x 8cm in size, exhibited some mobility. Nonetheless, its boundaries with adjacent structures were not clearly defined.

**Figure 1:**
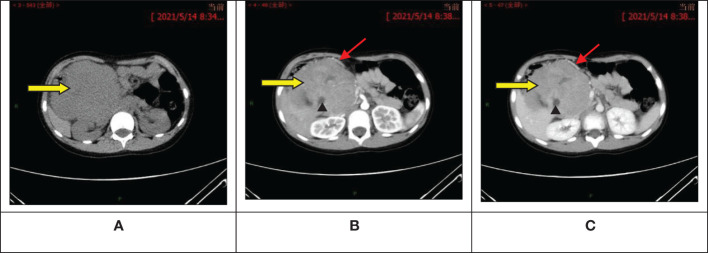
Abdominal computed tomography scan + enhancement show a primarily solid, mixed-density mass in the pancreatic head area, with significant enhancement of the solid component, interspersed low-attenuation foci, and visible tortuous small vessels within. **(A)** Pre-contrast phase; **(B)** Artery stage; **(C)** Portal vein stage. 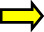: Tumor; 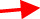: Visible tortuous small vessels; 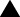: Interspersed low-attenuation foci.

**Figure 2:**
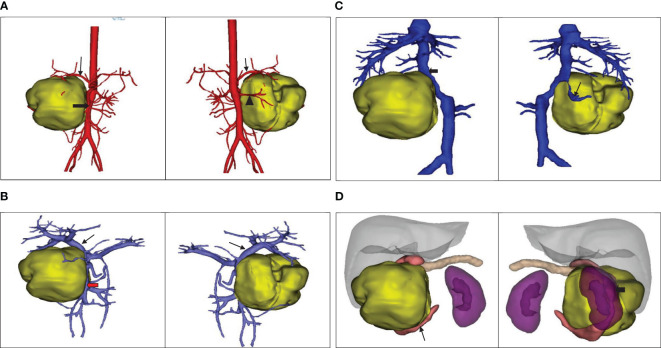
**(A)** The positional relationship between the tumor and the artery. ↓: Celiac trunk; ➨: Superior mesenteric artery; ▲: Right renal artery. **(B)** The positional relationship between the tumor and the portal vein. ↓: Portal vein; 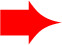:Superior mesenteric vein. **(C)** The tumor’s positional relationship with the inferior vena cava and right renal vein.↓: Right renal vein; ➨: Inferior vena cava. **(D)** The positional relationship between the tumor and the adjacent organs. 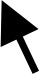: Duodenum; ➨: Right kidney.

The ultrasound examination revealed a heterogeneous echoic mass in the right upper abdomen, displaying nodular changes in certain parts of the tumor. Blood vessels traversing the mass were visible. The tumor had a close relationship with the head of the pancreas. The Computed Tomography(CT) scan showed a mixed-density mass in the pancreatic head area, primarily solid, with areas of low attenuation within. The mass was lobulated in shape, approximately 11.5cmx10.3cmx9.0cm in size. Upon enhancement, the mass demonstrated heterogeneous enhancement, with the solid component significantly enhanced, interspersed with a few low-attenuation foci, and tortuous small vessels could be seen within the mass (**Figures 1A–C**). No metastatic lesions were detected in the lungs or brain. The bone marrow smear, liver and kidney function tests, complete blood count, and coagulation parameters showed no abnormalities. The values of the serum tumor markers neuron-specific enolase (NSE), carcinoembryonic antigen(CEA), alpha-fetoprotein(AFP), lactate dehydrogenase (LDH), and carcinoma antigen 199 (CA199) were within normal limits. To further clarify the relationship between the tumor and surrounding blood vessels and organs, we employed a volume rendering technique (VR) for tumor assessment, as seen in Video 1. This technique also enabled individual analysis and assessment of the arteries, veins, and organs surrounding the tumor, facilitating an evaluation of the tumor’s resectability (**Figures 2A–D**).

**Figure 3:**
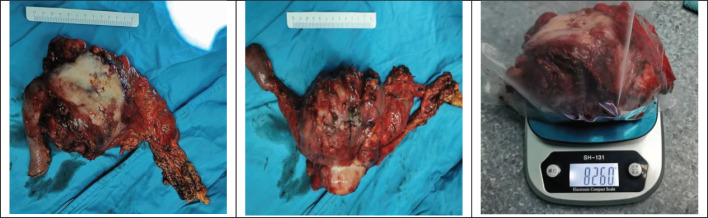
Postoperative gross specimen.

**Figure 4:**
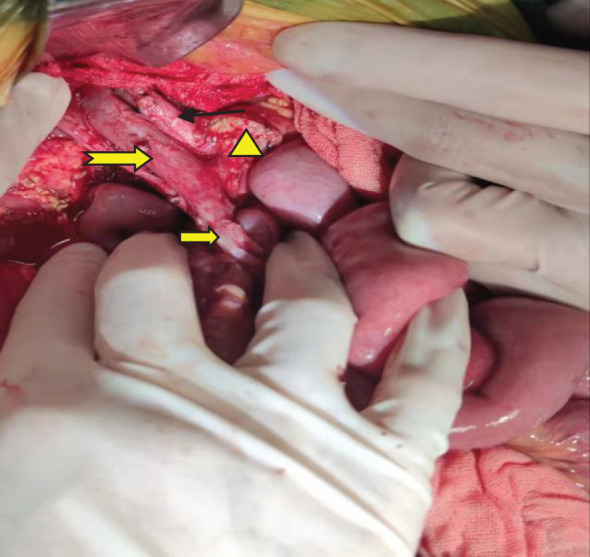
Gross resection of the visible tumor. 
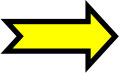
: Portal vein; 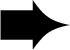: Common hepatic artery. 
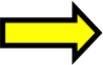
: Superior mesenteric vein. 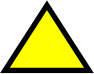
The pancreatic cut surface after tumor resection.

A pancreaticoduodenectomy and partial transverse colonectomy were successfully performed on the child. Intraoperative findings revealed a large tumor, approximately 12 cm x 10 cm x 11 cm, originating from the head of the pancreas and extending to the right middle lower abdomen. It had invaded about 2/3 of the transverse colon near the hepatic flexure and compressed the superior mesenteric vein (SMV). We excised the suspiciously invaded right wall of the SMV and reconstructed it using a continuous suturing technique with a 5-0 polypropylene surgical suture. No obvious peritoneal metastases were observed. In addition, we also noticed that the posterior edge of the tumor was pushing against the right kidney, and the right renal vein was deformed by the compression of the tumor. The postoperative gross specimen is depicted in **Figure 3**. All visible tumor within the operative field was excised during the surgical procedure (**Figure 4**). The tumor was characterized as a small round cell malignancy through an intraoperative frozen section procedure. However, the exact histological type of the tumor remained undetermined.

**Figure 5:**
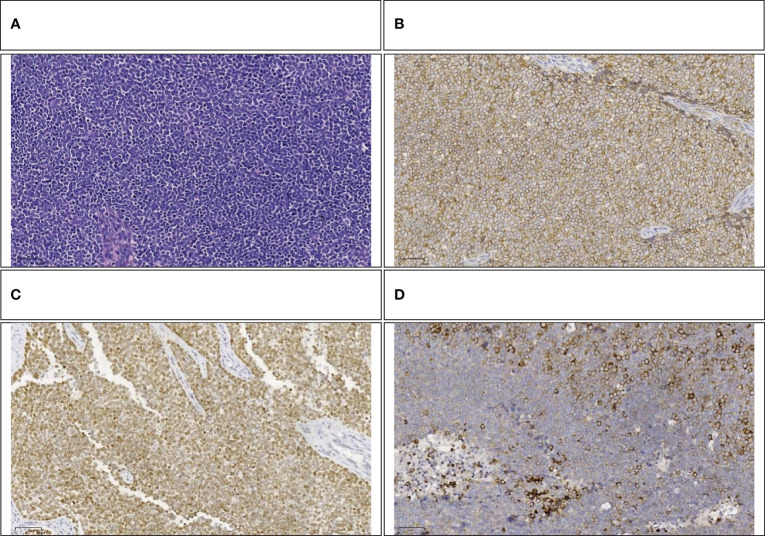
**(A)** Small round-cell malignant tumor (HE,100×). **(B)** Positive immunohistochemical staining for CD99(100×). **(C)** Positive immunohistochemical staining for NKX2.2 (100×). **(D)** Positive immunohistochemical staining for Syn (100×).

The pathology report from Fudan University Shanghai Cancer Center confirmed that the tumor, located in the head of the pancreas, was identified as Ewing’s sarcoma (**Figure 5A**) and invaded the full thickness of the small intestine and colon. Additionally, the presence of a vascular tumor thrombus was noted, along with the infiltration of tumor cells into the omental fibro-fatty tissue. The excised right wall of the superior mesenteric vein showed no signs of tumor invasion. No neoplastic activity was observed at the surgical margins of the gastric antrum, small intestine, and colon. The immunohistochemical assay revealed positivity for Vim, CD99(**Figure 5B**), NKX2.2(**Figure 5C**), SE (partially), Syn(**Figure 5D**), CgA (small focus), Ki-67 (approximately 60% in hotspot areas), INI-1, B-catenin (cytomembrane), and CK, negativity for Desmin, Myogenin, MyoD1, CD3, CD20, CD45, EMA, WwT1, Fli-1, and ERG. To further substantiate the diagnosis, we forwarded the specimens to Kang Shengda Medical Laboratory in Wuhan for comprehensive gene testing of pediatric solid tumors. The conducted test identified a breakpoint in the EWSR1 gene at chr22:29684161 and the FLI1 gene at chr11:128676663, resulting in an EWSR1-FLI1 fusion. Consequently, the final diagnosis confirmed that the patient had pancreatic Ewing’s sarcoma.

The pediatric patient experienced a smooth postoperative recovery and was transferred to the Hematology-Oncology department on the 15th day after surgery. Comprehensive evaluations, which included bone marrow aspiration and biopsy, postoperative fluorodeoxyglucose (FDG)-PET, cerebrospinal fluid analysis, abdominal enhanced Magnetic Resonance Imaging (MRI), enhanced CT, cranial and full-spine MRI, as well as color sonographic examination of both the urogenital and cardiac systems, and an ultrasound of the superficial lymph nodes, were performed. Collectively, these examinations confirmed the absence of distant metastasis. Subsequently, the patient underwent seven alternating cycles of VDC and IE chemotherapy. Upon reassessment, there were no signs of tumor recurrence, resulting in the cessation of the chemotherapy regimen. Following a period of 2 years and 6 months of regular follow-up examinations, the patient consistently demonstrated a sustained state of remission with no recurrence of the tumor.

Informed consent was obtained from the patient’s parents for the reporting of this case.

## Discussion

Ewing Sarcoma, recognized as the second most common malignant bone tumor in children and young adults, is typically identified in both bone and soft tissue. However, Extraskeletal Ewing Sarcoma, which exhibits a widespread anatomical distribution, can also sporadically appear in visceral organs such as the pancreas (9, 10). The presence of these tumors in the pancreas creates a distinct diagnostic dilemma due to the dominance of pancreatoblastoma, solid-pseudopapillary tumors, adenocarcinomas, and neuroendocrine tumors (11).

After conducting a comprehensive search and review of the literature, we discovered that, to date, there have been 44 reported cases of pancreatic Ewing’s sarcoma (1, 2, 5–7, 12–41), as shown in **Table 1**. This count also includes the case from the current study. It appears that the authors, Achufusi TG and Ahmad M, may have reported the same patient’s case from different perspectives (28, 32). Consequently, there would be only 43 reported cases of pancreatic Ewing’s sarcoma. Literature reports suggest that patients with pancreatic Ewing’s sarcoma typically have no specific clinical manifestations and often seek medical consultation due to upper abdominal pain. It occasionally presents as paraneoplastic syndromes such as precocious puberty, exophthalmos, and diabetes. However, jaundice is not a common manifestation. Upon discovery, the tumor frequently presents with a large size and is in an advanced stage, exhibiting local invasion or metastasis to the surrounding tissues and organs. At times, it is uncovered in that the tumor has invaded other organs, causing corresponding symptoms. Therefore, early diagnosis is quite difficult. In this case, the pediatric patient sought medical attention due to an unintentionally found abdominal lump, without experiencing any significant discomfort, corroborating the literature report. Surprisingly, despite the large size of the tumor, jaundice is not commonly observed, a marked contrast to adult pancreatic cancer where obstructive jaundice tends to appear early on. This may be attributed to several factors, which encompass the more uniform distribution of tumors across the gland, their infrequent origin from the ductal epithelium, and the distinct expansive growth pattern of these tumors, which contrasts with the infiltrative pattern often seen in ductal adenocarcinoma of the pancreas (21).

**Table 1:** 44 cases of Ewing sarcoma in the pancreas: demographics and clinical features.

Publication	Age	Gender	size(cm)	Location	Signs/symptoms	Treatment	Follow-up (months)	Outcome
Danner DB, 1994 (27)	17	M	Ukn	Head	UAQpain	Whipple	33	AWD
Lüttges J, 1997 (41)	13	F	2.2×8×10	Body	Dyspepsia,exophthalmus	operation,CT	2	AWD
Lüttges J, 1997 (41)	31	M	Ukn	Body	UAQpain	Biopsy,CT,operation	Ukn	Ukn
Bülchmann G, 2000 (31)	6	F	6×5×5	Head	UAQpain	Whipple and transverse Colon Resection	6	DOD
O’Sullivan MJ, 2001 (39)	20	M	3.5	Head	Ukn	Whipple	30	AWD
Gemechu T,2002 (20)	17	M	Ukn	Body	Abdominal swelling	Ukn	36	AWD
Movahedi-Lankarani S, 2002 (33)	21	F	Ukn	Head	Abdominal pain	Whipple	0	DOC
Movahedi-Lankarani S, 2002 (33)	25	F	Ukn	Head	Abdominal pain	Biopsy	Ukn	Ukn
Movahedi-Lankarani S, 2002 (33)	25	F	8	Head	Jaundice,abdominal pain	Biopsy	Ukn	Ukn
Movahedi-Lankarani S, 2002 (33)	6	M	3.5	Head	Jaundice,abdominal pain	Whipple,CT	48	DOD
Movahedi-Lankarani S, 2002 (33)	13	M	6	Head	Abdominal pain	Biopsy	43	AWD
Movahedi-Lankarani S, 2002 (33)	20	M	3.5	Head	Jaundice,abdominal pain	Whipple,CT(Ukn)	27	AWD
Movahedi-Lankarani S, 2002 (33)	17	M	9	Head	Jaundice,abdominalpain	Whipple,CT	33	AWD
Shorter NA, 2002 (21)	5	M	Ukn	Head	Jaundice	Whipple,CT	48	DOD
Shorter NA, 2002 (21)	12	M	Ukn	tail	Ukn	DP,CT	72	AWD
Takeuchi M, 2003 (40)	10	F	Ukn	Body	UAQpain	Ukn	3	DOD
Perek S, 2003 (30)	31	M	10×12	Head	UAQpain	Whipple,Recurrent mass resection with right kidney,CT,Metastatic lung wedge resection,CT	50	DOD
Schutte WP, 2006 (24)	2	F	6×4	Body	Precocious puberty	DP,CT	12	AWD
Welsch T, 2006 (18)	33	M	18×18×16	Body and tail	Nausea,vomiting	Partial gastrectomy and DPS,CT,Resection of liver metastases,Autologous stem cell transplantation	12	AWD
Doi H, 2009 (29)	37	M	4.2	Head	Jaundice	FNA,Whipple and a hepatic metastasis resection,CT,RT,ablation	10	AD
Menon BS, 2009 (2)	8	F	10 ×6×10	Body	UAQpain,Precocious Puberty	Bisopy,CT,RT	19	DOC
Jing H, 2011 (13)	24	F	Ukn	Uncinate process	None	tumor resection,RT,CT,Resection of recurrent tumor and the invaded tissue,CT	30	AD
Wakao J, 2011 (14)	3	M	8.2×6.5×10	Head	UAQpain and swelling	Biopsy,CT,Whipple	8	AWD
Maxwell L, 2011 (38)	11	M	9.8×7.8×6.4	Head	Anemia	Biopsy,CT,Whipple	Ukn	Ukn
Tortorelli AP 2012 (19)	18	M	33×27×18	Body and tail	Abdominal pain,exophthalmos	Total gastrectomy with DPS and Transverse Colon Resection;CT	9	AWD
Bose P, 2012 (17)	35	F	3	Body	Gallstone,pancreatitis	DPS and cholecystectomy	16	AWD
Reilly C, 2013 (26)	23	M	5.6 × 5.0 × 3.0	Body	UAQpain,nausea	Laparoscopic DPS	Ukn	Ukn
Jayant K, 2013 (22)	20	F	11 × 9	Body and tail	UAQpain	DPS,CT, RT	24	DOD
Mao Y, 2013 (7)	13	F	15×15×10	Head	UAQpain,diabetes mellitus	Resection of the uncinate process,CT,RT	41	DOD
Dias AR, 2013 (37)	25	F	4×3×2.5	Head	UAQpain	Whipple,CT	8	AWD
Kim JY, 2014 (25)	58	F	Ukn	Body	None	Bisopy,CT	Ukn	Ukn
Changal KH, 2014 (34)	60	M	3×3	Head	Abdominal pain	FNA,Biopsy,CT,Operation	Ukn	Ukn
Teixeira U, 2015 (6)	28	F	13×9×13	Head and body	UAQpain,Jaundice	Whipple	36	AWD
Kumar DN, 2015 (20)	22	M	20×15	Head	Abdominal pain	FNA,Whipple,CT	3	AWD
Nishizawa N, 2015 (12)	22	M	8	Head	UAQpain,nausea,vomiting	FNA,PPPD,CT,RT	12	AWD
Golhar A, 2017 (1)	17	F	5.6×7.4	Head and uncinate	Jaundice,itching	FNA,Whipple;CT,RT	6	AWD
Saif MW, 2017 (16)	38	F	8×10	Body	Abdominal pain	DPS;CT	6	AWD
Komforti MK, 2018 (15)	39	M	8×5.8	Body	Abdominal pain	FNA,Bisopy	1	AD
Yohannan B, 2020 (5)	26	F	10×9×7	Body and tail	left upper quadrant abdominal pain	FNA,CT,”DPS, subtotal gastrectomy, and left colectomy”,CT	9	DOD
Ahmad M, 2020 (32)	61	M	18.5×11×20	Head	abdominal pain,nausea	FNA,Pancreatic Tumour Excision, Cholecystectomy and Distal gastrectomy	Ukn	Ukn
Pinheiro RN, 2020 (36)	22	M	14	Head and body	abdominal pain	Resection: Pancreatic Body/Tail, Spleen, Omentum, Gastric Antrum/Body (Roux-en-Y), Hepatic III, Gallbladder;CT;RT	96	AWD
Achufusi TG, 2021 (28)	61	M	Ukn	Head	abdominal pain	FNA,Pancreatic Tumour Excision, Cholecystectomy and Distal gastrectomy	0.33	DOD
Liu Y-C, 2022 (35)	16	M	8.0 × 5.3× 9.3	Tail	left lower quadrant abdominal pain and dysuria	Biopsy,CT,tumor resection and left nephrectomy and left partial adrenalectomy,CT,RT	48	AWD
Present case	8	M	12×11×10	Head	None	Whipple and Partial transverse colectomy,CT	20	AWD

ES is recognized as a locally invasive tumor exhibiting aggressive growth patterns that are simultaneously infiltrative and expansive. These characteristics concur with the findings of numerous studies (42–44). Ewing’s sarcoma often presents with ill-defined margins and tends to displace surrounding organs (45). Although lymph node involvement in Ewing’s sarcoma cases is relatively infrequent, reported in merely 17.6%, the occurrence of distant metastases is common (46). Radiological findings often show the tumors to be iso-dense with regions of necrosis on pre-contrast CT images. These tumors typically appear iso-intense or hypo-intense on T1-weighted images (T1WI) and display heterogeneous hyper-intensity on T2-weighted images (T2WI) (45). Calcification is a rare phenomenon in Ewing’s sarcoma, observed in less than 10% of the cases. Typically, Ewing’s sarcoma presents as a large soft tissue mass, often exceeding 5 cm in size. One of the imaging characteristics of pancreatic Ewing’s sarcoma could be the absence of the typical double duct sign, indicative of pancreatic head cancer, even when the tumor is large (43, 45, 47).

We used the VR technique to assess the anatomical relationship between the tumor and surrounding blood vessels and organs. The assessment indicated that the tumor was located in the head of the pancreas, causing compression on the right kidney. It also suggested that the superior mesenteric vein was compressed and deformed, but not directly invaded by the tumor. This was confirmed by intraoperative findings that the superior mesenteric vein, despite being noticeably compressed, could be easily separated from the tumor. However, to achieve R0 resection, we excised part of the lateral wall of the superior mesenteric vein that was directly compressed by the tumor. Postoperative pathology confirmed that the removed mesenteric vascular wall was not invaded by the tumor, a characteristic that contrasts with pancreatic cancer which often directly invades surrounding blood vessels. This suggests that surgical resection for pancreatic Ewing’s sarcoma should not be readily dismissed merely based on preoperative imaging indicating vascular invasion or intraoperative findings of the tumor compressing or invading surrounding blood vessels. Some studies indicate that FDG PET-CT can potentially detect metastases in bone marrow and could potentially replace invasive procedures like bone marrow aspirates and trephine biopsies (11, 48). Regrettably, this specific examination was not conducted on the patient before the surgery. However, a comprehensive PET-CT scan was performed as part of a systematic evaluation before chemotherapy, which ruled out the presence of distant metastasis, serving to rectify the preoperative evaluation’s inadequacies.

The essential diagnostic criteria of ES include small round cell morphology and CD99 membranous expression, whereas the detection of FET-ETS fusion forms the desirable diagnostic criteria (3). Based on these criteria, the diagnosis of Pancreatic Ewing’s Sarcoma for the pediatric patient is confirmed. The standard treatment for newly identified Ewing sarcoma integrates multi-agent cytotoxic chemotherapy with local control strategies which include surgery and/or radiotherapy (49). Induction chemotherapy is necessary given the large tumor size and micrometastases (10). The approach to diagnosing and treating extraskeletal Ewing sarcoma (ES) follows the same guidelines as those implemented for bone ES (49). Despite the well-defined therapeutic principles, challenges persist in the diagnostic and therapeutic process for this specific case. For instance, the intraoperative frozen section analysis for this case revealed a small round-cell malignant tumor. The potential diagnoses could involve lymphoma, neuroblastoma, neuroendocrine tumor, poorly differentiated carcinoma, melanoma, or other round-cell sarcomas (5). The exact tumor type remained uncertain during the operation. Moreover, intraoperative exploration indicated that the tumor had no abdominal metastasis and appeared to be potentially resectable. We were then confronted with two choices. The first option entailed solely performing a biopsy, choosing induction chemotherapy based on the final pathological diagnose, and then conducting radical surgery. The primary advantage of the approach is an increased likelihood of achieving R0 resection, potentially eliminating the need for combined colectomy and superior mesenteric vein sidewall resection, and thereby reducing surgical risk and disability rate. However, a tumor biopsy could potentially lead to tumor dissemination, thus advancing the cancer stage and necessitating the child patient to endure the trauma and pain of two surgeries. The second approach involves one-stage radical tumor resection. For the case, this would entail not only performing a traditional pancreatoduodenectomy but also conducting a resection of part of the transverse colon. One major drawback of this approach lies in its high risk and aggressive nature, especially when the specific pathological type remains uncertain. Should postoperative pathology confirm diseases, such as lymphoma, which are treatable with chemotherapy (50), the consequent trauma inflicted on the child would be excessive.

Considering that open biopsy is quite traumatic and may increase the risk of tumor staging and dissemination, preoperative fine needle aspiration (FNA) has become a crucial diagnostic procedure for pediatric solid tumors. Therefore, we have compiled the FNA examination results of these 43 cases of pancreatic Ewing’s sarcoma. **Table 2** presents the FNA results of the eight patients who underwent preoperative FNA examination. Various studies advocate FNA as the initial diagnostic method for patients with pancreatic mass. The diagnostic step carries utmost significance as it guides the treatment regimen and enhances the likelihood of a favorable outcome (51–53). However, FNA harbors certain limitations. For instance, it may fail to procure sufficient tissue necessary for a comprehensive diagnosis, particularly for tests necessitating larger tissue samples. Additionally, the results obtained from FNA can occasionally be inconclusive or ambiguous, mandating open abdominal biopsy to reach a definitive diagnosis. Of the 43 cases of pancreatic Ewing’s sarcoma summarized in this article, eight had FNA examinations. However, only one case was confirmed as pancreatic Ewing’s sarcoma through FNA. Two cases were negative, one was misdiagnosed as a pancreatic endocrine tumor, one was diagnosed as a neuroendocrine tumor metastasized to the liver, and one was suspected of pancreatic neuroectodermal tumor, but could not be confirmed. Another two cases were diagnosed as small round cell tumors. Although the diagnosis rate is not high, the value of FNA is expected to increase with advancements in medical technology. In our report, given the unique exophytic growth pattern and the substantial size of the tumor that was palpable under the skin, the pediatric patient should ideally have undergone an FNA examination prior to surgery. This procedure could have potentially facilitated a precise preoperative diagnosis.

**Table 2:** 8 cases FNA Examination Results of Ewing sarcoma in the pancreas.

Publication	FNA Examination Results
Achufusi TG,2021 (28)	no evidence of malignancy
Yohannan B, 2020 (5)	extraosseous ES of the pancreas
Komforti MK,2018 (15)	scant cellularity and rare atypical single cells
Golhar A,2017 (1)	a pancreatic neuroendocrine neoplasm was suspected
Nishizawa N, 2015 (12)	PNET was suspected, a definitive diagnosis was difficult because of inadequate samples
Kumar DN, 2015 (20)	undifferentiated small round cell malignant tumor
Changal KH, 2014 (34)	A histopathologic possibility of round cell tumour was made
Doi H, 2009 (29)	a metastatic carcinoma or poorly differentiated endocrine carcinoma was suspected

Studies have identified specific predictors for improved survival, such as younger age at diagnosis and complete tumor resections. Conversely, factors including age over 14 years, primary tumor volume exceeding 200 cc, and the occurrence of bone marrow or lung metastases have been recognized as major risk factors for a poorer prognosis (54, 55). The pediatric patient has been followed up for two and a half years with no recurrence of the tumor and has exhibited satisfactory growth and development. Favorable prognostic factors for this patient include a younger age at diagnosis, the absence of distant metastases, and complete tumor resection. The large tumor volume, visible tumor thrombus in the vessels, and tumor invasion into the omentum are considered adverse factors. All invaded tissues and organs were completely resected, which explains the decision not to administer postoperative radiation therapy to the patient. Despite the favorable therapeutic outcome in this case, our diagnostic and treatment process had its shortcomings. For example, we did not sufficiently recognize the necessity of preoperative whole-body PET CT scans and tumor FNA procedures. Through the study of this case and related literature, we have deepened our understanding of rare tumors in children. Given the paramount goal of optimizing the patient’s survival benefit, it is essential to refer such rare cases of pediatric pancreatic tumors to specialized centers dedicated to pediatric solid tumor treatment.

## Conclusion

ES can originate in the pancreas and is highly aggressive. Early and accurate diagnosis is crucial for effective management of these tumors. Aggressive surgical resection, alongside multimodal therapy, can potentially improve prognosis. However, the prognosis for this rare malignancy remains unsatisfactory. Therefore, further case accumulation and investigation are required to improve outcomes.

## Data availability statement

The original contributions presented in the study are included in the article/**Supplementary Materials**, further inquiries can be directed to the corresponding author/s.

## Ethics statement

The studies involving humans were approved by Ethics Committee of the Children’s Hospital of Anhui Province. The studies were conducted in accordance with the local legislation and institutional requirements. Written informed consent for participation was not required from the participants or the participants’ legal guardians/next of kin in accordance with the national legislation and institutional requirements. Written informed consent was obtained from the individual(s) for the publication of any potentially identifiable images or data included in this article.

## Author contributions

ZL: Writing – original draft, Writing – review & editing. JB: Conceptualization, Data curation, Supervision, Writing – review & editing. YY: Methodology, Visualization, Writing – review & editing. DW: Data curation, Formal analysis, Writing – review & editing. SQ: Conceptualization, Project administration, Writing – review & editing.
